# Supplementary figures and images for: ADID-UNET—a segmentation model for COVID-19 infection from lung CT scans (part 2 of 2)
Source: PeerJ Comput Sci. 2021 Jan 26;7:e349. doi: 10.7717/peerj-cs.349 (PMC7924694; doi:10.7717/peerj-cs.349)

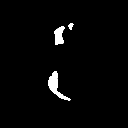

Supplement: Supplemental Information 1 [file peerj-cs-07-349-s001.zip › Upload_Code/ADID-UNET/results/Test_Image_190_Predict.png]

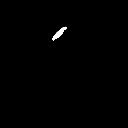

Supplement: Supplemental Information 1 [file peerj-cs-07-349-s001.zip › Upload_Code/ADID-UNET/results/Test_Image_191_Predict.png]

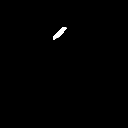

Supplement: Supplemental Information 1 [file peerj-cs-07-349-s001.zip › Upload_Code/ADID-UNET/results/Test_Image_192_Predict.png]

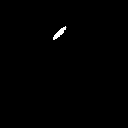

Supplement: Supplemental Information 1 [file peerj-cs-07-349-s001.zip › Upload_Code/ADID-UNET/results/Test_Image_193_Predict.png]

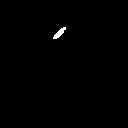

Supplement: Supplemental Information 1 [file peerj-cs-07-349-s001.zip › Upload_Code/ADID-UNET/results/Test_Image_194_Predict.png]

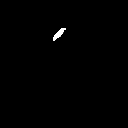

Supplement: Supplemental Information 1 [file peerj-cs-07-349-s001.zip › Upload_Code/ADID-UNET/results/Test_Image_195_Predict.png]

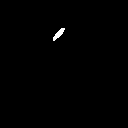

Supplement: Supplemental Information 1 [file peerj-cs-07-349-s001.zip › Upload_Code/ADID-UNET/results/Test_Image_196_Predict.png]

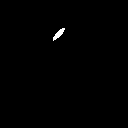

Supplement: Supplemental Information 1 [file peerj-cs-07-349-s001.zip › Upload_Code/ADID-UNET/results/Test_Image_197_Predict.png]

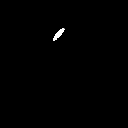

Supplement: Supplemental Information 1 [file peerj-cs-07-349-s001.zip › Upload_Code/ADID-UNET/results/Test_Image_198_Predict.png]

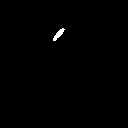

Supplement: Supplemental Information 1 [file peerj-cs-07-349-s001.zip › Upload_Code/ADID-UNET/results/Test_Image_199_Predict.png]

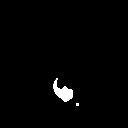

Supplement: Supplemental Information 1 [file peerj-cs-07-349-s001.zip › Upload_Code/ADID-UNET/results/Test_Image_2_Predict.png]

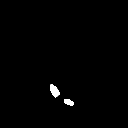

Supplement: Supplemental Information 1 [file peerj-cs-07-349-s001.zip › Upload_Code/ADID-UNET/results/Test_Image_20_Predict.png]

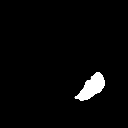

Supplement: Supplemental Information 1 [file peerj-cs-07-349-s001.zip › Upload_Code/ADID-UNET/results/Test_Image_200_Predict.png]

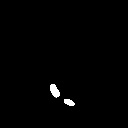

Supplement: Supplemental Information 1 [file peerj-cs-07-349-s001.zip › Upload_Code/ADID-UNET/results/Test_Image_21_Predict.png]

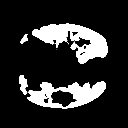

Supplement: Supplemental Information 1 [file peerj-cs-07-349-s001.zip › Upload_Code/ADID-UNET/results/Test_Image_22_Predict.png]

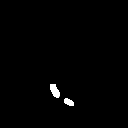

Supplement: Supplemental Information 1 [file peerj-cs-07-349-s001.zip › Upload_Code/ADID-UNET/results/Test_Image_23_Predict.png]

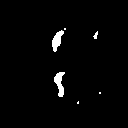

Supplement: Supplemental Information 1 [file peerj-cs-07-349-s001.zip › Upload_Code/ADID-UNET/results/Test_Image_24_Predict.png]

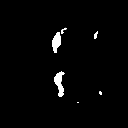

Supplement: Supplemental Information 1 [file peerj-cs-07-349-s001.zip › Upload_Code/ADID-UNET/results/Test_Image_25_Predict.png]

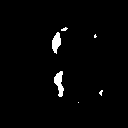

Supplement: Supplemental Information 1 [file peerj-cs-07-349-s001.zip › Upload_Code/ADID-UNET/results/Test_Image_26_Predict.png]

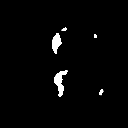

Supplement: Supplemental Information 1 [file peerj-cs-07-349-s001.zip › Upload_Code/ADID-UNET/results/Test_Image_27_Predict.png]

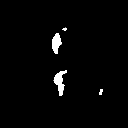

Supplement: Supplemental Information 1 [file peerj-cs-07-349-s001.zip › Upload_Code/ADID-UNET/results/Test_Image_28_Predict.png]

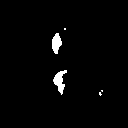

Supplement: Supplemental Information 1 [file peerj-cs-07-349-s001.zip › Upload_Code/ADID-UNET/results/Test_Image_29_Predict.png]

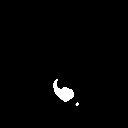

Supplement: Supplemental Information 1 [file peerj-cs-07-349-s001.zip › Upload_Code/ADID-UNET/results/Test_Image_3_Predict.png]

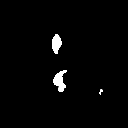

Supplement: Supplemental Information 1 [file peerj-cs-07-349-s001.zip › Upload_Code/ADID-UNET/results/Test_Image_30_Predict.png]

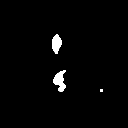

Supplement: Supplemental Information 1 [file peerj-cs-07-349-s001.zip › Upload_Code/ADID-UNET/results/Test_Image_31_Predict.png]

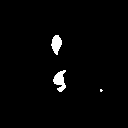

Supplement: Supplemental Information 1 [file peerj-cs-07-349-s001.zip › Upload_Code/ADID-UNET/results/Test_Image_32_Predict.png]

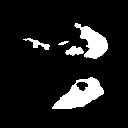

Supplement: Supplemental Information 1 [file peerj-cs-07-349-s001.zip › Upload_Code/ADID-UNET/results/Test_Image_33_Predict.png]

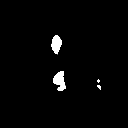

Supplement: Supplemental Information 1 [file peerj-cs-07-349-s001.zip › Upload_Code/ADID-UNET/results/Test_Image_34_Predict.png]

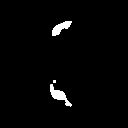

Supplement: Supplemental Information 1 [file peerj-cs-07-349-s001.zip › Upload_Code/ADID-UNET/results/Test_Image_35_Predict.png]

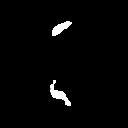

Supplement: Supplemental Information 1 [file peerj-cs-07-349-s001.zip › Upload_Code/ADID-UNET/results/Test_Image_36_Predict.png]

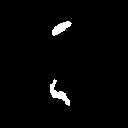

Supplement: Supplemental Information 1 [file peerj-cs-07-349-s001.zip › Upload_Code/ADID-UNET/results/Test_Image_37_Predict.png]

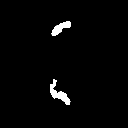

Supplement: Supplemental Information 1 [file peerj-cs-07-349-s001.zip › Upload_Code/ADID-UNET/results/Test_Image_38_Predict.png]

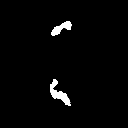

Supplement: Supplemental Information 1 [file peerj-cs-07-349-s001.zip › Upload_Code/ADID-UNET/results/Test_Image_39_Predict.png]

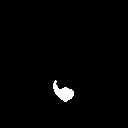

Supplement: Supplemental Information 1 [file peerj-cs-07-349-s001.zip › Upload_Code/ADID-UNET/results/Test_Image_4_Predict.png]

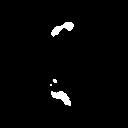

Supplement: Supplemental Information 1 [file peerj-cs-07-349-s001.zip › Upload_Code/ADID-UNET/results/Test_Image_40_Predict.png]

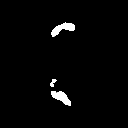

Supplement: Supplemental Information 1 [file peerj-cs-07-349-s001.zip › Upload_Code/ADID-UNET/results/Test_Image_41_Predict.png]

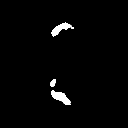

Supplement: Supplemental Information 1 [file peerj-cs-07-349-s001.zip › Upload_Code/ADID-UNET/results/Test_Image_42_Predict.png]

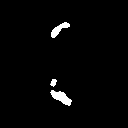

Supplement: Supplemental Information 1 [file peerj-cs-07-349-s001.zip › Upload_Code/ADID-UNET/results/Test_Image_43_Predict.png]

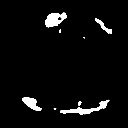

Supplement: Supplemental Information 1 [file peerj-cs-07-349-s001.zip › Upload_Code/ADID-UNET/results/Test_Image_44_Predict.png]

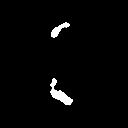

Supplement: Supplemental Information 1 [file peerj-cs-07-349-s001.zip › Upload_Code/ADID-UNET/results/Test_Image_45_Predict.png]

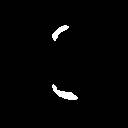

Supplement: Supplemental Information 1 [file peerj-cs-07-349-s001.zip › Upload_Code/ADID-UNET/results/Test_Image_46_Predict.png]

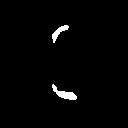

Supplement: Supplemental Information 1 [file peerj-cs-07-349-s001.zip › Upload_Code/ADID-UNET/results/Test_Image_47_Predict.png]

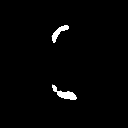

Supplement: Supplemental Information 1 [file peerj-cs-07-349-s001.zip › Upload_Code/ADID-UNET/results/Test_Image_48_Predict.png]

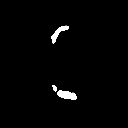

Supplement: Supplemental Information 1 [file peerj-cs-07-349-s001.zip › Upload_Code/ADID-UNET/results/Test_Image_49_Predict.png]

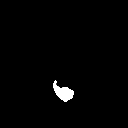

Supplement: Supplemental Information 1 [file peerj-cs-07-349-s001.zip › Upload_Code/ADID-UNET/results/Test_Image_5_Predict.png]

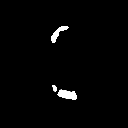

Supplement: Supplemental Information 1 [file peerj-cs-07-349-s001.zip › Upload_Code/ADID-UNET/results/Test_Image_50_Predict.png]

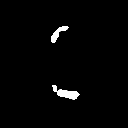

Supplement: Supplemental Information 1 [file peerj-cs-07-349-s001.zip › Upload_Code/ADID-UNET/results/Test_Image_51_Predict.png]

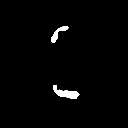

Supplement: Supplemental Information 1 [file peerj-cs-07-349-s001.zip › Upload_Code/ADID-UNET/results/Test_Image_52_Predict.png]

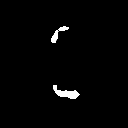

Supplement: Supplemental Information 1 [file peerj-cs-07-349-s001.zip › Upload_Code/ADID-UNET/results/Test_Image_53_Predict.png]

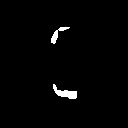

Supplement: Supplemental Information 1 [file peerj-cs-07-349-s001.zip › Upload_Code/ADID-UNET/results/Test_Image_54_Predict.png]

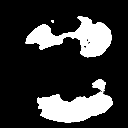

Supplement: Supplemental Information 1 [file peerj-cs-07-349-s001.zip › Upload_Code/ADID-UNET/results/Test_Image_55_Predict.png]

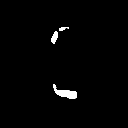

Supplement: Supplemental Information 1 [file peerj-cs-07-349-s001.zip › Upload_Code/ADID-UNET/results/Test_Image_56_Predict.png]

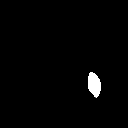

Supplement: Supplemental Information 1 [file peerj-cs-07-349-s001.zip › Upload_Code/ADID-UNET/results/Test_Image_57_Predict.png]

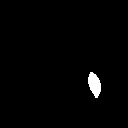

Supplement: Supplemental Information 1 [file peerj-cs-07-349-s001.zip › Upload_Code/ADID-UNET/results/Test_Image_58_Predict.png]

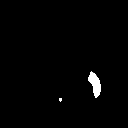

Supplement: Supplemental Information 1 [file peerj-cs-07-349-s001.zip › Upload_Code/ADID-UNET/results/Test_Image_59_Predict.png]

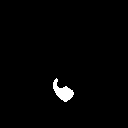

Supplement: Supplemental Information 1 [file peerj-cs-07-349-s001.zip › Upload_Code/ADID-UNET/results/Test_Image_6_Predict.png]

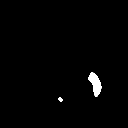

Supplement: Supplemental Information 1 [file peerj-cs-07-349-s001.zip › Upload_Code/ADID-UNET/results/Test_Image_60_Predict.png]

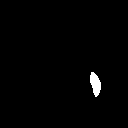

Supplement: Supplemental Information 1 [file peerj-cs-07-349-s001.zip › Upload_Code/ADID-UNET/results/Test_Image_61_Predict.png]

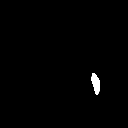

Supplement: Supplemental Information 1 [file peerj-cs-07-349-s001.zip › Upload_Code/ADID-UNET/results/Test_Image_62_Predict.png]

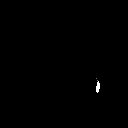

Supplement: Supplemental Information 1 [file peerj-cs-07-349-s001.zip › Upload_Code/ADID-UNET/results/Test_Image_63_Predict.png]

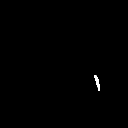

Supplement: Supplemental Information 1 [file peerj-cs-07-349-s001.zip › Upload_Code/ADID-UNET/results/Test_Image_64_Predict.png]

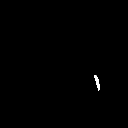

Supplement: Supplemental Information 1 [file peerj-cs-07-349-s001.zip › Upload_Code/ADID-UNET/results/Test_Image_65_Predict.png]

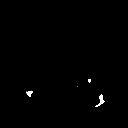

Supplement: Supplemental Information 1 [file peerj-cs-07-349-s001.zip › Upload_Code/ADID-UNET/results/Test_Image_66_Predict.png]

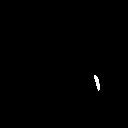

Supplement: Supplemental Information 1 [file peerj-cs-07-349-s001.zip › Upload_Code/ADID-UNET/results/Test_Image_67_Predict.png]

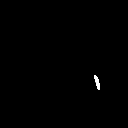

Supplement: Supplemental Information 1 [file peerj-cs-07-349-s001.zip › Upload_Code/ADID-UNET/results/Test_Image_68_Predict.png]

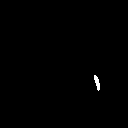

Supplement: Supplemental Information 1 [file peerj-cs-07-349-s001.zip › Upload_Code/ADID-UNET/results/Test_Image_69_Predict.png]

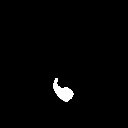

Supplement: Supplemental Information 1 [file peerj-cs-07-349-s001.zip › Upload_Code/ADID-UNET/results/Test_Image_7_Predict.png]

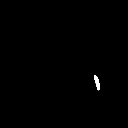

Supplement: Supplemental Information 1 [file peerj-cs-07-349-s001.zip › Upload_Code/ADID-UNET/results/Test_Image_70_Predict.png]

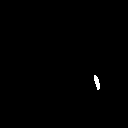

Supplement: Supplemental Information 1 [file peerj-cs-07-349-s001.zip › Upload_Code/ADID-UNET/results/Test_Image_71_Predict.png]

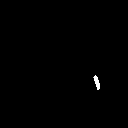

Supplement: Supplemental Information 1 [file peerj-cs-07-349-s001.zip › Upload_Code/ADID-UNET/results/Test_Image_72_Predict.png]

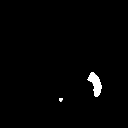

Supplement: Supplemental Information 1 [file peerj-cs-07-349-s001.zip › Upload_Code/ADID-UNET/results/Test_Image_73_Predict.png]

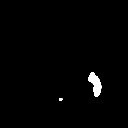

Supplement: Supplemental Information 1 [file peerj-cs-07-349-s001.zip › Upload_Code/ADID-UNET/results/Test_Image_74_Predict.png]

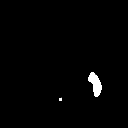

Supplement: Supplemental Information 1 [file peerj-cs-07-349-s001.zip › Upload_Code/ADID-UNET/results/Test_Image_75_Predict.png]

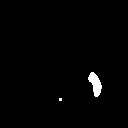

Supplement: Supplemental Information 1 [file peerj-cs-07-349-s001.zip › Upload_Code/ADID-UNET/results/Test_Image_76_Predict.png]

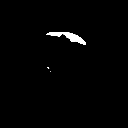

Supplement: Supplemental Information 1 [file peerj-cs-07-349-s001.zip › Upload_Code/ADID-UNET/results/Test_Image_77_Predict.png]

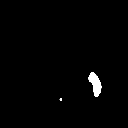

Supplement: Supplemental Information 1 [file peerj-cs-07-349-s001.zip › Upload_Code/ADID-UNET/results/Test_Image_78_Predict.png]

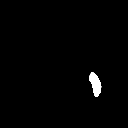

Supplement: Supplemental Information 1 [file peerj-cs-07-349-s001.zip › Upload_Code/ADID-UNET/results/Test_Image_79_Predict.png]

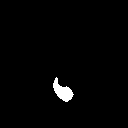

Supplement: Supplemental Information 1 [file peerj-cs-07-349-s001.zip › Upload_Code/ADID-UNET/results/Test_Image_8_Predict.png]

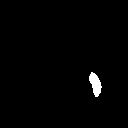

Supplement: Supplemental Information 1 [file peerj-cs-07-349-s001.zip › Upload_Code/ADID-UNET/results/Test_Image_80_Predict.png]

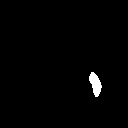

Supplement: Supplemental Information 1 [file peerj-cs-07-349-s001.zip › Upload_Code/ADID-UNET/results/Test_Image_81_Predict.png]

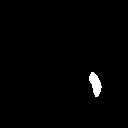

Supplement: Supplemental Information 1 [file peerj-cs-07-349-s001.zip › Upload_Code/ADID-UNET/results/Test_Image_82_Predict.png]

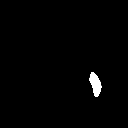

Supplement: Supplemental Information 1 [file peerj-cs-07-349-s001.zip › Upload_Code/ADID-UNET/results/Test_Image_83_Predict.png]

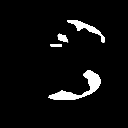

Supplement: Supplemental Information 1 [file peerj-cs-07-349-s001.zip › Upload_Code/ADID-UNET/results/Test_Image_84_Predict.png]

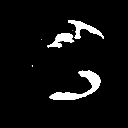

Supplement: Supplemental Information 1 [file peerj-cs-07-349-s001.zip › Upload_Code/ADID-UNET/results/Test_Image_85_Predict.png]

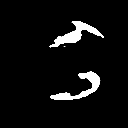

Supplement: Supplemental Information 1 [file peerj-cs-07-349-s001.zip › Upload_Code/ADID-UNET/results/Test_Image_86_Predict.png]

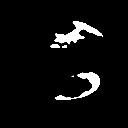

Supplement: Supplemental Information 1 [file peerj-cs-07-349-s001.zip › Upload_Code/ADID-UNET/results/Test_Image_87_Predict.png]

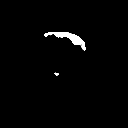

Supplement: Supplemental Information 1 [file peerj-cs-07-349-s001.zip › Upload_Code/ADID-UNET/results/Test_Image_88_Predict.png]

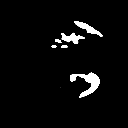

Supplement: Supplemental Information 1 [file peerj-cs-07-349-s001.zip › Upload_Code/ADID-UNET/results/Test_Image_89_Predict.png]

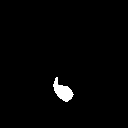

Supplement: Supplemental Information 1 [file peerj-cs-07-349-s001.zip › Upload_Code/ADID-UNET/results/Test_Image_9_Predict.png]

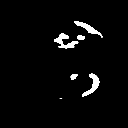

Supplement: Supplemental Information 1 [file peerj-cs-07-349-s001.zip › Upload_Code/ADID-UNET/results/Test_Image_90_Predict.png]

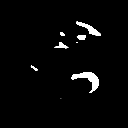

Supplement: Supplemental Information 1 [file peerj-cs-07-349-s001.zip › Upload_Code/ADID-UNET/results/Test_Image_91_Predict.png]

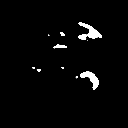

Supplement: Supplemental Information 1 [file peerj-cs-07-349-s001.zip › Upload_Code/ADID-UNET/results/Test_Image_92_Predict.png]

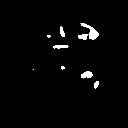

Supplement: Supplemental Information 1 [file peerj-cs-07-349-s001.zip › Upload_Code/ADID-UNET/results/Test_Image_93_Predict.png]

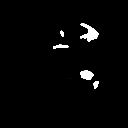

Supplement: Supplemental Information 1 [file peerj-cs-07-349-s001.zip › Upload_Code/ADID-UNET/results/Test_Image_94_Predict.png]

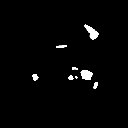

Supplement: Supplemental Information 1 [file peerj-cs-07-349-s001.zip › Upload_Code/ADID-UNET/results/Test_Image_95_Predict.png]

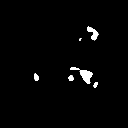

Supplement: Supplemental Information 1 [file peerj-cs-07-349-s001.zip › Upload_Code/ADID-UNET/results/Test_Image_96_Predict.png]

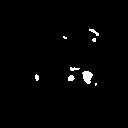

Supplement: Supplemental Information 1 [file peerj-cs-07-349-s001.zip › Upload_Code/ADID-UNET/results/Test_Image_97_Predict.png]

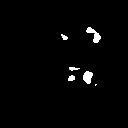

Supplement: Supplemental Information 1 [file peerj-cs-07-349-s001.zip › Upload_Code/ADID-UNET/results/Test_Image_98_Predict.png]

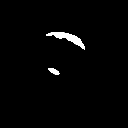

Supplement: Supplemental Information 1 [file peerj-cs-07-349-s001.zip › Upload_Code/ADID-UNET/results/Test_Image_99_Predict.png]
