# Supplementary figures and images for: ADID-UNET—a segmentation model for COVID-19 infection from lung CT scans (part 1 of 2)
Source: PeerJ Comput Sci. 2021 Jan 26;7:e349. doi: 10.7717/peerj-cs.349 (PMC7924694; doi:10.7717/peerj-cs.349)

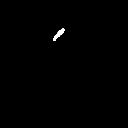

Supplement: Supplemental Information 1 [file peerj-cs-07-349-s001.zip › Upload_Code/ADID-UNET/results/Test_Image_1_Predict.png]

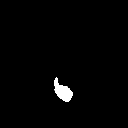

Supplement: Supplemental Information 1 [file peerj-cs-07-349-s001.zip › Upload_Code/ADID-UNET/results/Test_Image_10_Predict.png]

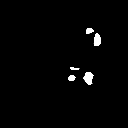

Supplement: Supplemental Information 1 [file peerj-cs-07-349-s001.zip › Upload_Code/ADID-UNET/results/Test_Image_100_Predict.png]

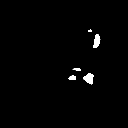

Supplement: Supplemental Information 1 [file peerj-cs-07-349-s001.zip › Upload_Code/ADID-UNET/results/Test_Image_101_Predict.png]

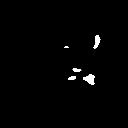

Supplement: Supplemental Information 1 [file peerj-cs-07-349-s001.zip › Upload_Code/ADID-UNET/results/Test_Image_102_Predict.png]

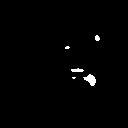

Supplement: Supplemental Information 1 [file peerj-cs-07-349-s001.zip › Upload_Code/ADID-UNET/results/Test_Image_103_Predict.png]

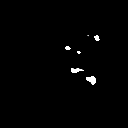

Supplement: Supplemental Information 1 [file peerj-cs-07-349-s001.zip › Upload_Code/ADID-UNET/results/Test_Image_104_Predict.png]

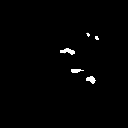

Supplement: Supplemental Information 1 [file peerj-cs-07-349-s001.zip › Upload_Code/ADID-UNET/results/Test_Image_105_Predict.png]

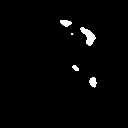

Supplement: Supplemental Information 1 [file peerj-cs-07-349-s001.zip › Upload_Code/ADID-UNET/results/Test_Image_106_Predict.png]

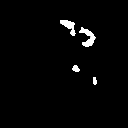

Supplement: Supplemental Information 1 [file peerj-cs-07-349-s001.zip › Upload_Code/ADID-UNET/results/Test_Image_107_Predict.png]

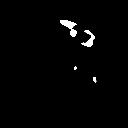

Supplement: Supplemental Information 1 [file peerj-cs-07-349-s001.zip › Upload_Code/ADID-UNET/results/Test_Image_108_Predict.png]

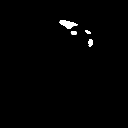

Supplement: Supplemental Information 1 [file peerj-cs-07-349-s001.zip › Upload_Code/ADID-UNET/results/Test_Image_109_Predict.png]

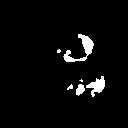

Supplement: Supplemental Information 1 [file peerj-cs-07-349-s001.zip › Upload_Code/ADID-UNET/results/Test_Image_11_Predict.png]

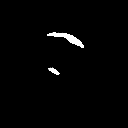

Supplement: Supplemental Information 1 [file peerj-cs-07-349-s001.zip › Upload_Code/ADID-UNET/results/Test_Image_110_Predict.png]

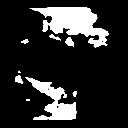

Supplement: Supplemental Information 1 [file peerj-cs-07-349-s001.zip › Upload_Code/ADID-UNET/results/Test_Image_111_Predict.png]

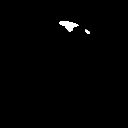

Supplement: Supplemental Information 1 [file peerj-cs-07-349-s001.zip › Upload_Code/ADID-UNET/results/Test_Image_112_Predict.png]

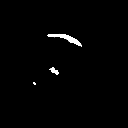

Supplement: Supplemental Information 1 [file peerj-cs-07-349-s001.zip › Upload_Code/ADID-UNET/results/Test_Image_113_Predict.png]

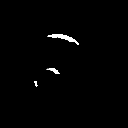

Supplement: Supplemental Information 1 [file peerj-cs-07-349-s001.zip › Upload_Code/ADID-UNET/results/Test_Image_114_Predict.png]

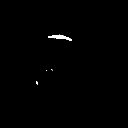

Supplement: Supplemental Information 1 [file peerj-cs-07-349-s001.zip › Upload_Code/ADID-UNET/results/Test_Image_115_Predict.png]

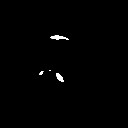

Supplement: Supplemental Information 1 [file peerj-cs-07-349-s001.zip › Upload_Code/ADID-UNET/results/Test_Image_116_Predict.png]

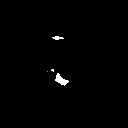

Supplement: Supplemental Information 1 [file peerj-cs-07-349-s001.zip › Upload_Code/ADID-UNET/results/Test_Image_117_Predict.png]

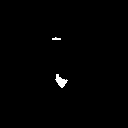

Supplement: Supplemental Information 1 [file peerj-cs-07-349-s001.zip › Upload_Code/ADID-UNET/results/Test_Image_118_Predict.png]

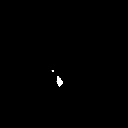

Supplement: Supplemental Information 1 [file peerj-cs-07-349-s001.zip › Upload_Code/ADID-UNET/results/Test_Image_119_Predict.png]

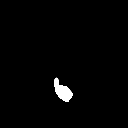

Supplement: Supplemental Information 1 [file peerj-cs-07-349-s001.zip › Upload_Code/ADID-UNET/results/Test_Image_12_Predict.png]

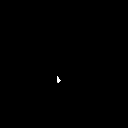

Supplement: Supplemental Information 1 [file peerj-cs-07-349-s001.zip › Upload_Code/ADID-UNET/results/Test_Image_120_Predict.png]

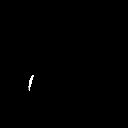

Supplement: Supplemental Information 1 [file peerj-cs-07-349-s001.zip › Upload_Code/ADID-UNET/results/Test_Image_121_Predict.png]

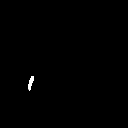

Supplement: Supplemental Information 1 [file peerj-cs-07-349-s001.zip › Upload_Code/ADID-UNET/results/Test_Image_122_Predict.png]

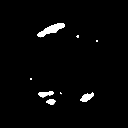

Supplement: Supplemental Information 1 [file peerj-cs-07-349-s001.zip › Upload_Code/ADID-UNET/results/Test_Image_123_Predict.png]

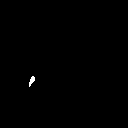

Supplement: Supplemental Information 1 [file peerj-cs-07-349-s001.zip › Upload_Code/ADID-UNET/results/Test_Image_124_Predict.png]

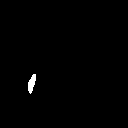

Supplement: Supplemental Information 1 [file peerj-cs-07-349-s001.zip › Upload_Code/ADID-UNET/results/Test_Image_125_Predict.png]

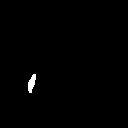

Supplement: Supplemental Information 1 [file peerj-cs-07-349-s001.zip › Upload_Code/ADID-UNET/results/Test_Image_126_Predict.png]

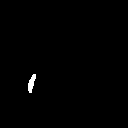

Supplement: Supplemental Information 1 [file peerj-cs-07-349-s001.zip › Upload_Code/ADID-UNET/results/Test_Image_127_Predict.png]

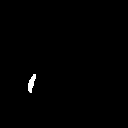

Supplement: Supplemental Information 1 [file peerj-cs-07-349-s001.zip › Upload_Code/ADID-UNET/results/Test_Image_128_Predict.png]

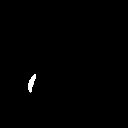

Supplement: Supplemental Information 1 [file peerj-cs-07-349-s001.zip › Upload_Code/ADID-UNET/results/Test_Image_129_Predict.png]

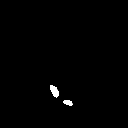

Supplement: Supplemental Information 1 [file peerj-cs-07-349-s001.zip › Upload_Code/ADID-UNET/results/Test_Image_13_Predict.png]

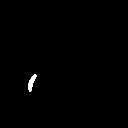

Supplement: Supplemental Information 1 [file peerj-cs-07-349-s001.zip › Upload_Code/ADID-UNET/results/Test_Image_130_Predict.png]

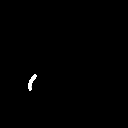

Supplement: Supplemental Information 1 [file peerj-cs-07-349-s001.zip › Upload_Code/ADID-UNET/results/Test_Image_131_Predict.png]

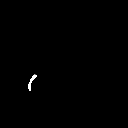

Supplement: Supplemental Information 1 [file peerj-cs-07-349-s001.zip › Upload_Code/ADID-UNET/results/Test_Image_132_Predict.png]

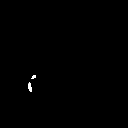

Supplement: Supplemental Information 1 [file peerj-cs-07-349-s001.zip › Upload_Code/ADID-UNET/results/Test_Image_133_Predict.png]

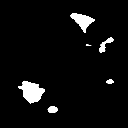

Supplement: Supplemental Information 1 [file peerj-cs-07-349-s001.zip › Upload_Code/ADID-UNET/results/Test_Image_134_Predict.png]

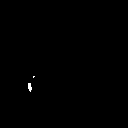

Supplement: Supplemental Information 1 [file peerj-cs-07-349-s001.zip › Upload_Code/ADID-UNET/results/Test_Image_135_Predict.png]

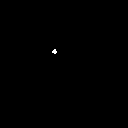

Supplement: Supplemental Information 1 [file peerj-cs-07-349-s001.zip › Upload_Code/ADID-UNET/results/Test_Image_136_Predict.png]

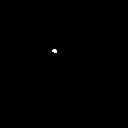

Supplement: Supplemental Information 1 [file peerj-cs-07-349-s001.zip › Upload_Code/ADID-UNET/results/Test_Image_137_Predict.png]

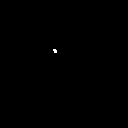

Supplement: Supplemental Information 1 [file peerj-cs-07-349-s001.zip › Upload_Code/ADID-UNET/results/Test_Image_138_Predict.png]

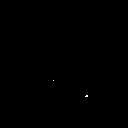

Supplement: Supplemental Information 1 [file peerj-cs-07-349-s001.zip › Upload_Code/ADID-UNET/results/Test_Image_139_Predict.png]

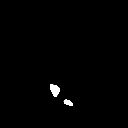

Supplement: Supplemental Information 1 [file peerj-cs-07-349-s001.zip › Upload_Code/ADID-UNET/results/Test_Image_14_Predict.png]

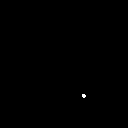

Supplement: Supplemental Information 1 [file peerj-cs-07-349-s001.zip › Upload_Code/ADID-UNET/results/Test_Image_140_Predict.png]

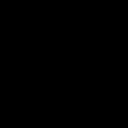

Supplement: Supplemental Information 1 [file peerj-cs-07-349-s001.zip › Upload_Code/ADID-UNET/results/Test_Image_141_Predict.png]

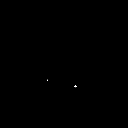

Supplement: Supplemental Information 1 [file peerj-cs-07-349-s001.zip › Upload_Code/ADID-UNET/results/Test_Image_142_Predict.png]

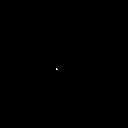

Supplement: Supplemental Information 1 [file peerj-cs-07-349-s001.zip › Upload_Code/ADID-UNET/results/Test_Image_143_Predict.png]

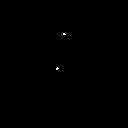

Supplement: Supplemental Information 1 [file peerj-cs-07-349-s001.zip › Upload_Code/ADID-UNET/results/Test_Image_144_Predict.png]

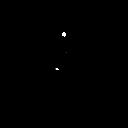

Supplement: Supplemental Information 1 [file peerj-cs-07-349-s001.zip › Upload_Code/ADID-UNET/results/Test_Image_146_Predict.png]

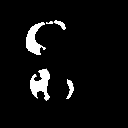

Supplement: Supplemental Information 1 [file peerj-cs-07-349-s001.zip › Upload_Code/ADID-UNET/results/Test_Image_147_Predict.png]

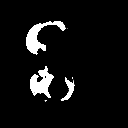

Supplement: Supplemental Information 1 [file peerj-cs-07-349-s001.zip › Upload_Code/ADID-UNET/results/Test_Image_148_Predict.png]

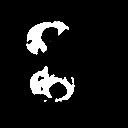

Supplement: Supplemental Information 1 [file peerj-cs-07-349-s001.zip › Upload_Code/ADID-UNET/results/Test_Image_149_Predict.png]

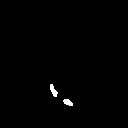

Supplement: Supplemental Information 1 [file peerj-cs-07-349-s001.zip › Upload_Code/ADID-UNET/results/Test_Image_15_Predict.png]

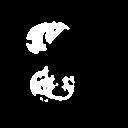

Supplement: Supplemental Information 1 [file peerj-cs-07-349-s001.zip › Upload_Code/ADID-UNET/results/Test_Image_150_Predict.png]

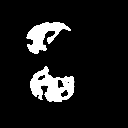

Supplement: Supplemental Information 1 [file peerj-cs-07-349-s001.zip › Upload_Code/ADID-UNET/results/Test_Image_151_Predict.png]

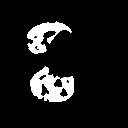

Supplement: Supplemental Information 1 [file peerj-cs-07-349-s001.zip › Upload_Code/ADID-UNET/results/Test_Image_152_Predict.png]

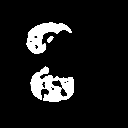

Supplement: Supplemental Information 1 [file peerj-cs-07-349-s001.zip › Upload_Code/ADID-UNET/results/Test_Image_153_Predict.png]

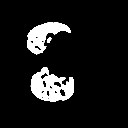

Supplement: Supplemental Information 1 [file peerj-cs-07-349-s001.zip › Upload_Code/ADID-UNET/results/Test_Image_154_Predict.png]

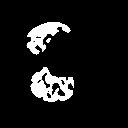

Supplement: Supplemental Information 1 [file peerj-cs-07-349-s001.zip › Upload_Code/ADID-UNET/results/Test_Image_155_Predict.png]

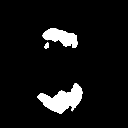

Supplement: Supplemental Information 1 [file peerj-cs-07-349-s001.zip › Upload_Code/ADID-UNET/results/Test_Image_156_Predict.png]

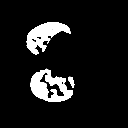

Supplement: Supplemental Information 1 [file peerj-cs-07-349-s001.zip › Upload_Code/ADID-UNET/results/Test_Image_157_Predict.png]

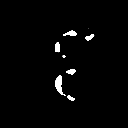

Supplement: Supplemental Information 1 [file peerj-cs-07-349-s001.zip › Upload_Code/ADID-UNET/results/Test_Image_158_Predict.png]

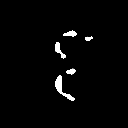

Supplement: Supplemental Information 1 [file peerj-cs-07-349-s001.zip › Upload_Code/ADID-UNET/results/Test_Image_159_Predict.png]

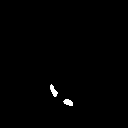

Supplement: Supplemental Information 1 [file peerj-cs-07-349-s001.zip › Upload_Code/ADID-UNET/results/Test_Image_16_Predict.png]

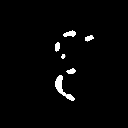

Supplement: Supplemental Information 1 [file peerj-cs-07-349-s001.zip › Upload_Code/ADID-UNET/results/Test_Image_160_Predict.png]

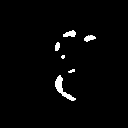

Supplement: Supplemental Information 1 [file peerj-cs-07-349-s001.zip › Upload_Code/ADID-UNET/results/Test_Image_161_Predict.png]

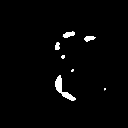

Supplement: Supplemental Information 1 [file peerj-cs-07-349-s001.zip › Upload_Code/ADID-UNET/results/Test_Image_162_Predict.png]

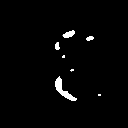

Supplement: Supplemental Information 1 [file peerj-cs-07-349-s001.zip › Upload_Code/ADID-UNET/results/Test_Image_163_Predict.png]

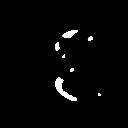

Supplement: Supplemental Information 1 [file peerj-cs-07-349-s001.zip › Upload_Code/ADID-UNET/results/Test_Image_164_Predict.png]

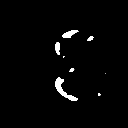

Supplement: Supplemental Information 1 [file peerj-cs-07-349-s001.zip › Upload_Code/ADID-UNET/results/Test_Image_165_Predict.png]

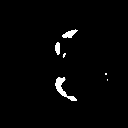

Supplement: Supplemental Information 1 [file peerj-cs-07-349-s001.zip › Upload_Code/ADID-UNET/results/Test_Image_166_Predict.png]

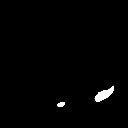

Supplement: Supplemental Information 1 [file peerj-cs-07-349-s001.zip › Upload_Code/ADID-UNET/results/Test_Image_167_Predict.png]

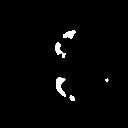

Supplement: Supplemental Information 1 [file peerj-cs-07-349-s001.zip › Upload_Code/ADID-UNET/results/Test_Image_168_Predict.png]

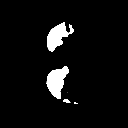

Supplement: Supplemental Information 1 [file peerj-cs-07-349-s001.zip › Upload_Code/ADID-UNET/results/Test_Image_169_Predict.png]

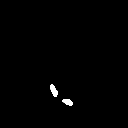

Supplement: Supplemental Information 1 [file peerj-cs-07-349-s001.zip › Upload_Code/ADID-UNET/results/Test_Image_17_Predict.png]

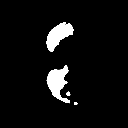

Supplement: Supplemental Information 1 [file peerj-cs-07-349-s001.zip › Upload_Code/ADID-UNET/results/Test_Image_170_Predict.png]

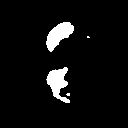

Supplement: Supplemental Information 1 [file peerj-cs-07-349-s001.zip › Upload_Code/ADID-UNET/results/Test_Image_171_Predict.png]

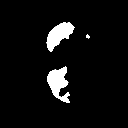

Supplement: Supplemental Information 1 [file peerj-cs-07-349-s001.zip › Upload_Code/ADID-UNET/results/Test_Image_172_Predict.png]

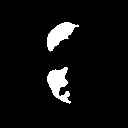

Supplement: Supplemental Information 1 [file peerj-cs-07-349-s001.zip › Upload_Code/ADID-UNET/results/Test_Image_173_Predict.png]

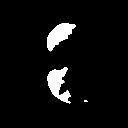

Supplement: Supplemental Information 1 [file peerj-cs-07-349-s001.zip › Upload_Code/ADID-UNET/results/Test_Image_174_Predict.png]

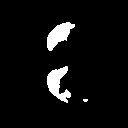

Supplement: Supplemental Information 1 [file peerj-cs-07-349-s001.zip › Upload_Code/ADID-UNET/results/Test_Image_175_Predict.png]

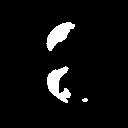

Supplement: Supplemental Information 1 [file peerj-cs-07-349-s001.zip › Upload_Code/ADID-UNET/results/Test_Image_176_Predict.png]

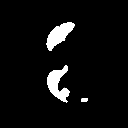

Supplement: Supplemental Information 1 [file peerj-cs-07-349-s001.zip › Upload_Code/ADID-UNET/results/Test_Image_177_Predict.png]

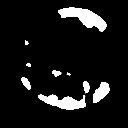

Supplement: Supplemental Information 1 [file peerj-cs-07-349-s001.zip › Upload_Code/ADID-UNET/results/Test_Image_178_Predict.png]

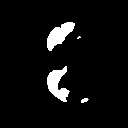

Supplement: Supplemental Information 1 [file peerj-cs-07-349-s001.zip › Upload_Code/ADID-UNET/results/Test_Image_179_Predict.png]

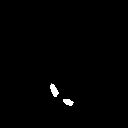

Supplement: Supplemental Information 1 [file peerj-cs-07-349-s001.zip › Upload_Code/ADID-UNET/results/Test_Image_18_Predict.png]

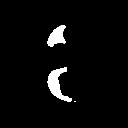

Supplement: Supplemental Information 1 [file peerj-cs-07-349-s001.zip › Upload_Code/ADID-UNET/results/Test_Image_180_Predict.png]

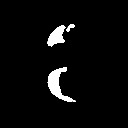

Supplement: Supplemental Information 1 [file peerj-cs-07-349-s001.zip › Upload_Code/ADID-UNET/results/Test_Image_181_Predict.png]

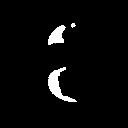

Supplement: Supplemental Information 1 [file peerj-cs-07-349-s001.zip › Upload_Code/ADID-UNET/results/Test_Image_182_Predict.png]

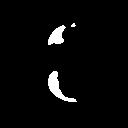

Supplement: Supplemental Information 1 [file peerj-cs-07-349-s001.zip › Upload_Code/ADID-UNET/results/Test_Image_183_Predict.png]

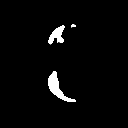

Supplement: Supplemental Information 1 [file peerj-cs-07-349-s001.zip › Upload_Code/ADID-UNET/results/Test_Image_184_Predict.png]

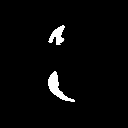

Supplement: Supplemental Information 1 [file peerj-cs-07-349-s001.zip › Upload_Code/ADID-UNET/results/Test_Image_185_Predict.png]

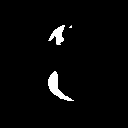

Supplement: Supplemental Information 1 [file peerj-cs-07-349-s001.zip › Upload_Code/ADID-UNET/results/Test_Image_186_Predict.png]

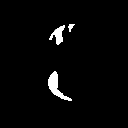

Supplement: Supplemental Information 1 [file peerj-cs-07-349-s001.zip › Upload_Code/ADID-UNET/results/Test_Image_187_Predict.png]

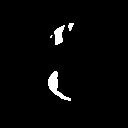

Supplement: Supplemental Information 1 [file peerj-cs-07-349-s001.zip › Upload_Code/ADID-UNET/results/Test_Image_188_Predict.png]

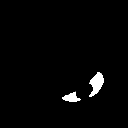

Supplement: Supplemental Information 1 [file peerj-cs-07-349-s001.zip › Upload_Code/ADID-UNET/results/Test_Image_189_Predict.png]

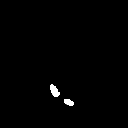

Supplement: Supplemental Information 1 [file peerj-cs-07-349-s001.zip › Upload_Code/ADID-UNET/results/Test_Image_19_Predict.png]
