# Supplementary material for: ADID-UNET—a segmentation model for COVID-19 infection from lung CT scans
Source: PeerJ Comput Sci. 2021 Jan 26;7:e349. doi: 10.7717/peerj-cs.349 (PMC7924694; doi:10.7717/peerj-cs.349)
Supplement: Supplemental Information 2 [file peerj-cs-07-349-s002.pdf]

ADID-Model Usage and Results Verification

Section 1: Dataset:

1. Datasets:

For training sets, validation sets, and test sets, original images, and labels are required. Example of the original picture and labels you need.

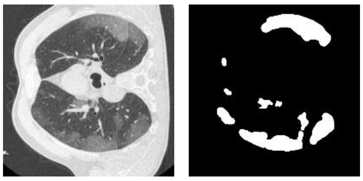

2. Code and Dataset and Results:

We've uploaded all the codes, datasets, and results as attachments to the PeerJ journal, which should look like this when you download all the folders.

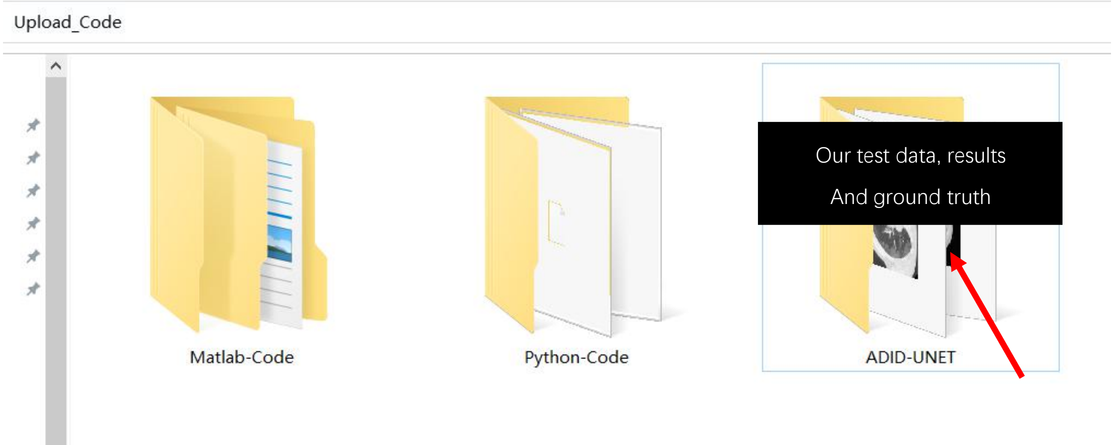

Next, We will explain the contents of each folder. The "Python-Code" folder mainly contains Python codes, as shown in the following figure:

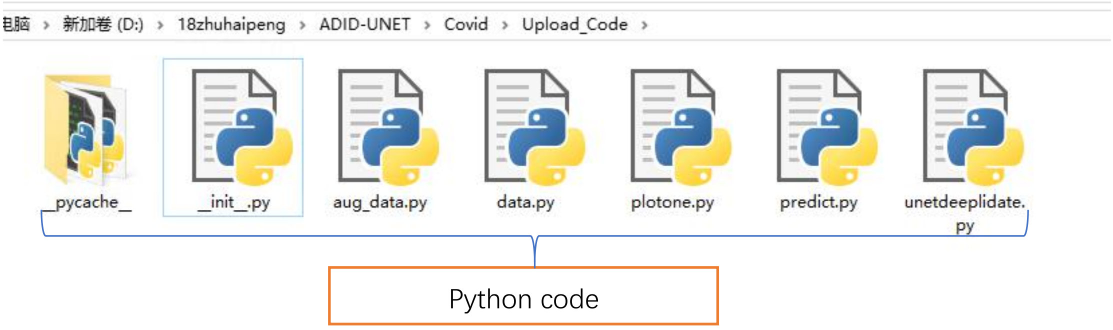

The "Matlab-Code" folder, contains "EvaluateResultas", "evaluation", "Results" and "TestingSet" four folders, as shown in the following figure. Also, we have explained the contents of each folder and how to run the code in Section 2.3.

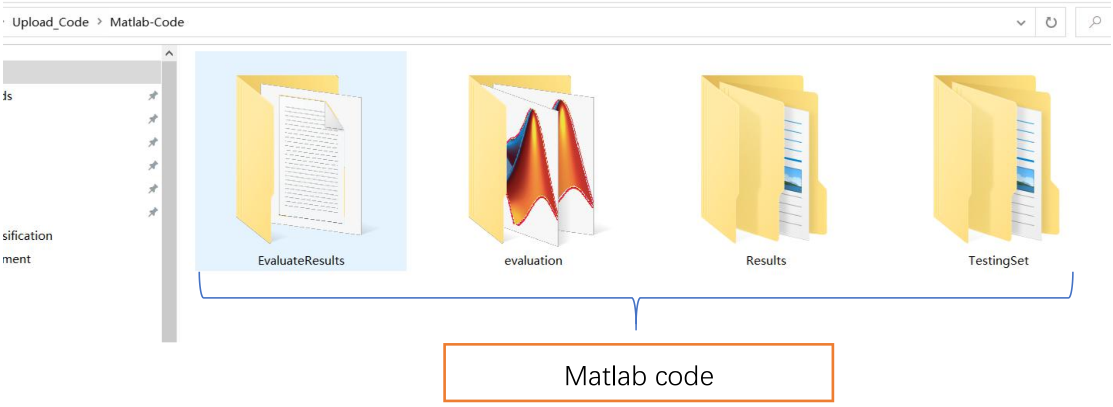

The "ADID-UNET folder includes the "test_inputs_images", "test_ground_truth" and "results" folders, as shown below:

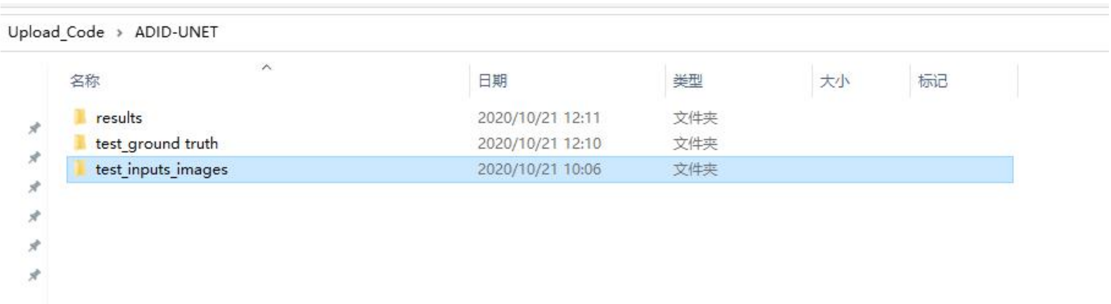

There are the contents of the "test_inputs_images", "test_ground_truth" and "results" folders:

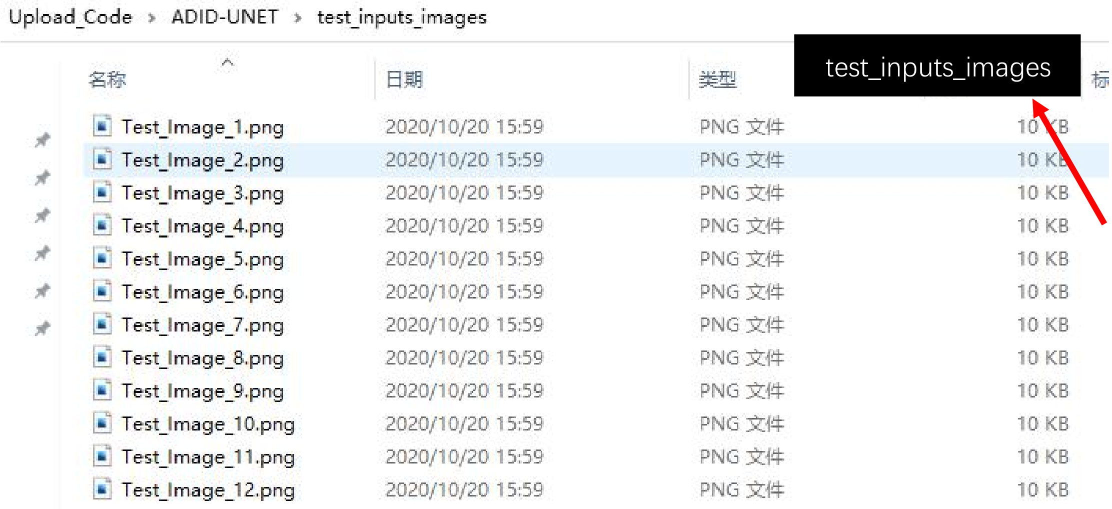

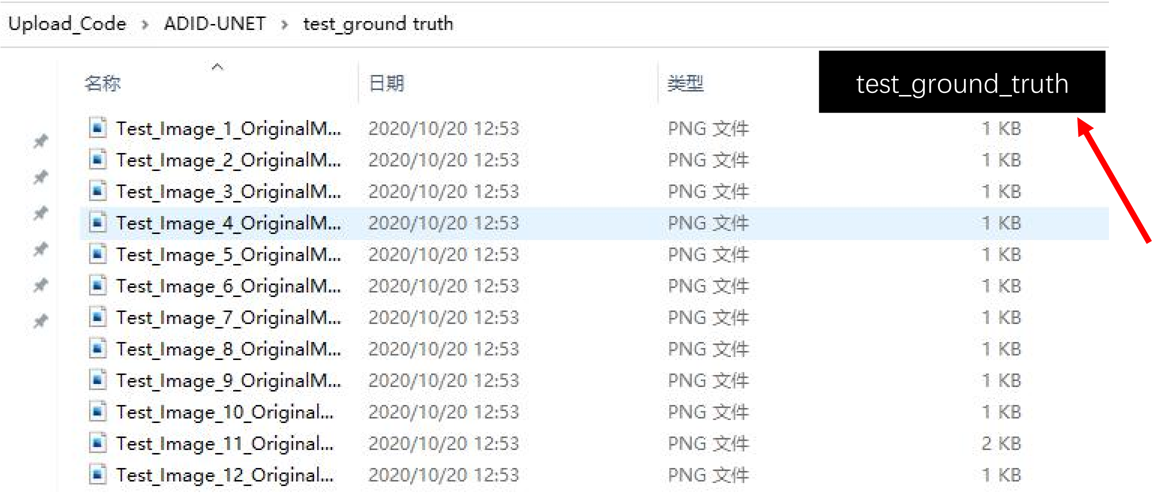

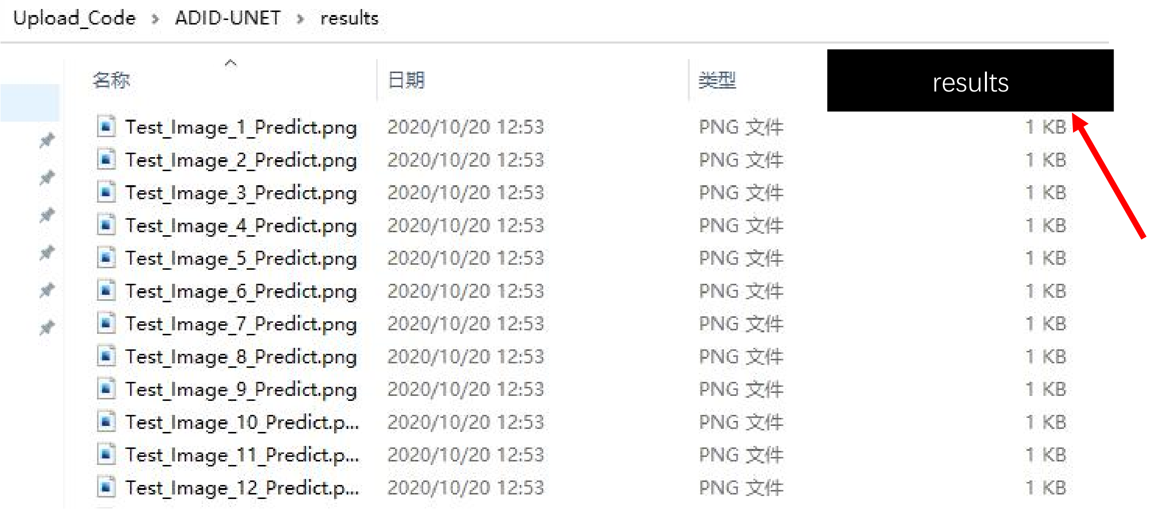

Section 2: Instruction for executing the codes:

Procedure Summary:

- Use **aug.py** to augment the data, and then use the **data.py** to convert the data to **.NPY** format.
- Use **unetdeeplidate.py** to train the ADID-UNET model.
- Use **predict.py** and **plotone.py** to obtain the prediction results and segmentation indicators, such as accuracy, precision, Dice coefficient, sensitivity, specificity and F1 score.
- Use **matlab script** to obtain the other three segmentation indexes, Structural metric (Sm), Enhance alignment metric (Eα), Mean Absolute Error (MAE).

THE FLOW CHART FOR TRAINING THE MODEL AND TESTING IT

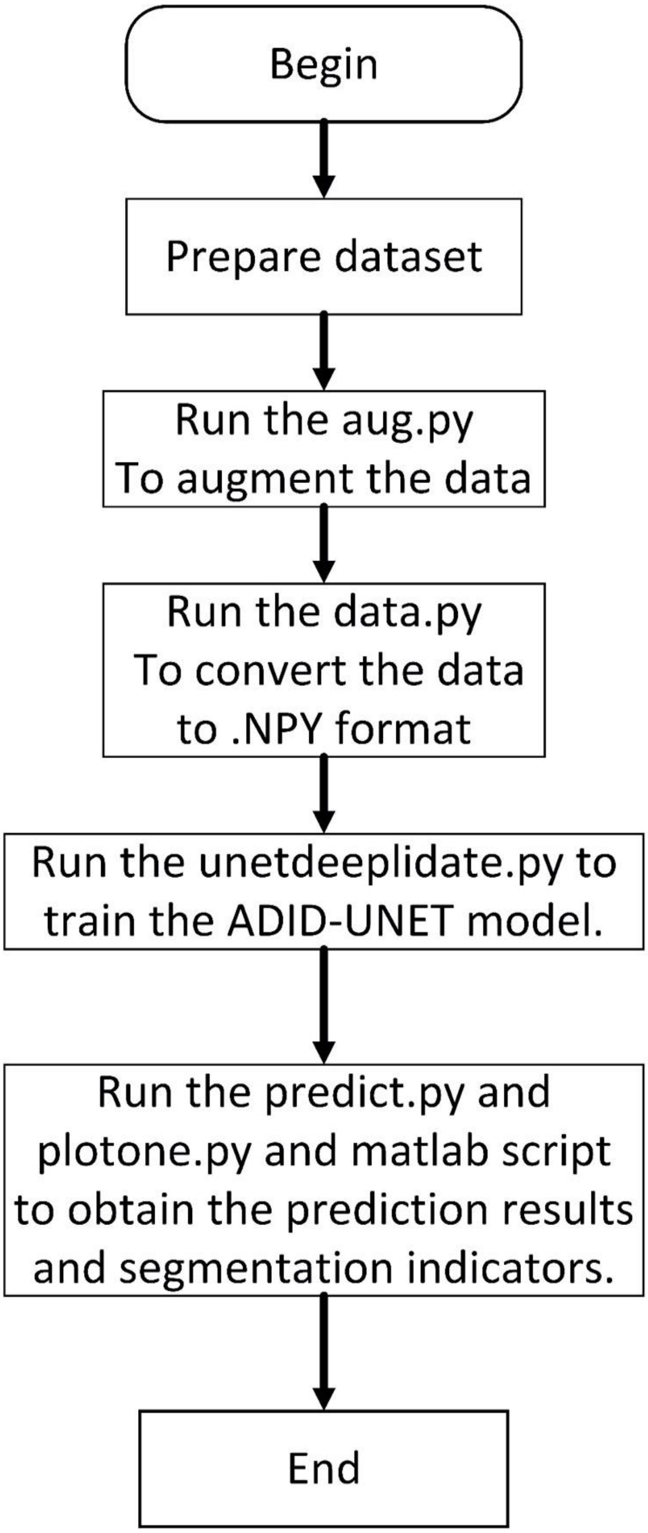

FLOW CHART FOR USING THE TRAINED MODEL

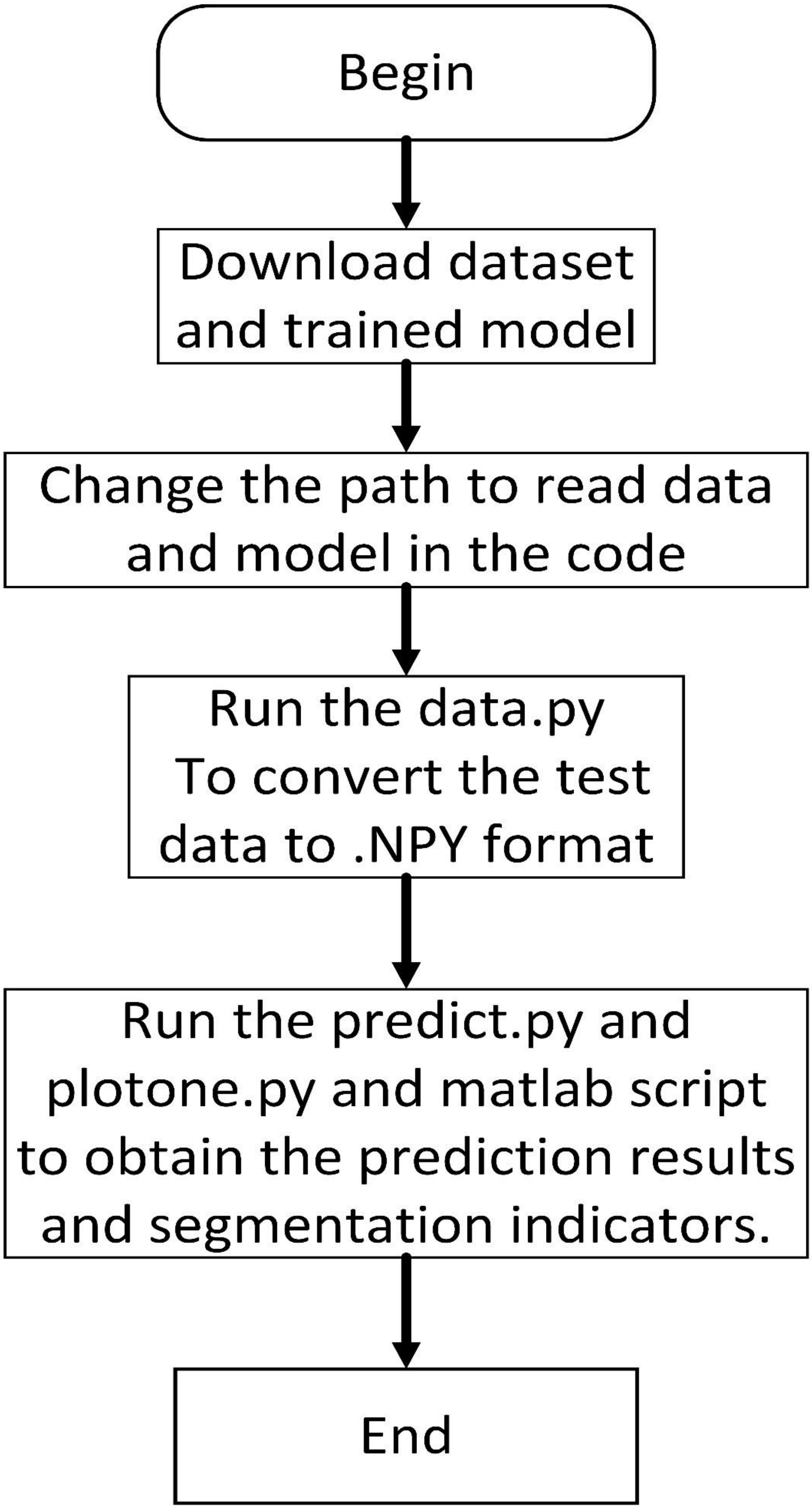

Detailed Information about the codes

2.1 Information about Python codes of the ADID-UNET model.

(1) Here, you need to build a “raw” file to store datasets, including training sets, validation sets, test sets, as well as automatically segmented images of models. As shown in the following figure:

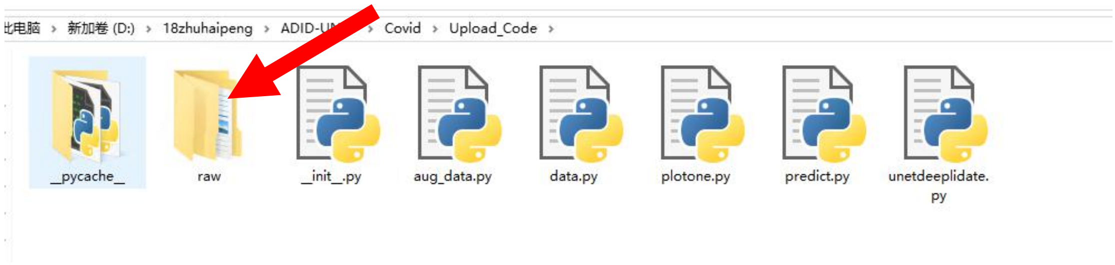

(2) Aug_data.py is mainly to expand training set images. The process is as follows.

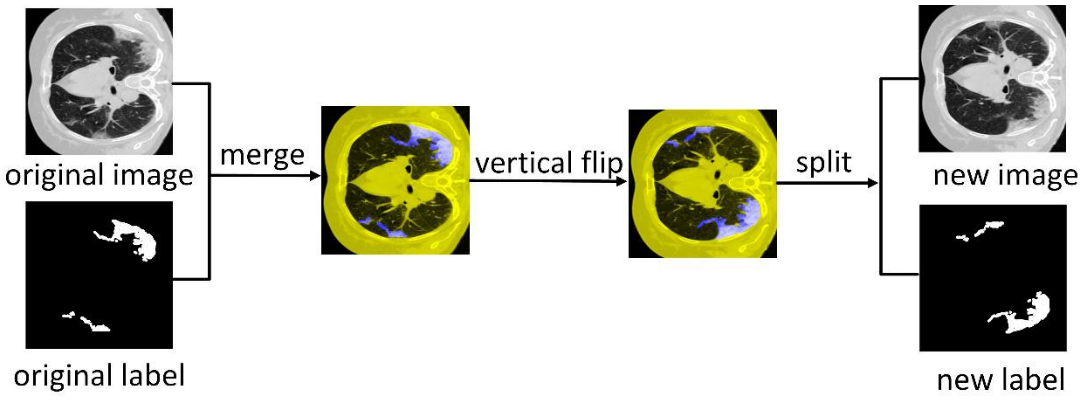

(3) Data.py is mainly about training set, validation set, and test set images saved as .NPY.

(4) Predict.py is used for obtaining the segmentation result of the model.

(5) Plotone.py is used for test AUC and ROC curve

(6) Unetdeeplidate.py is the ADID-UNET models code.

As shown in the following figure:

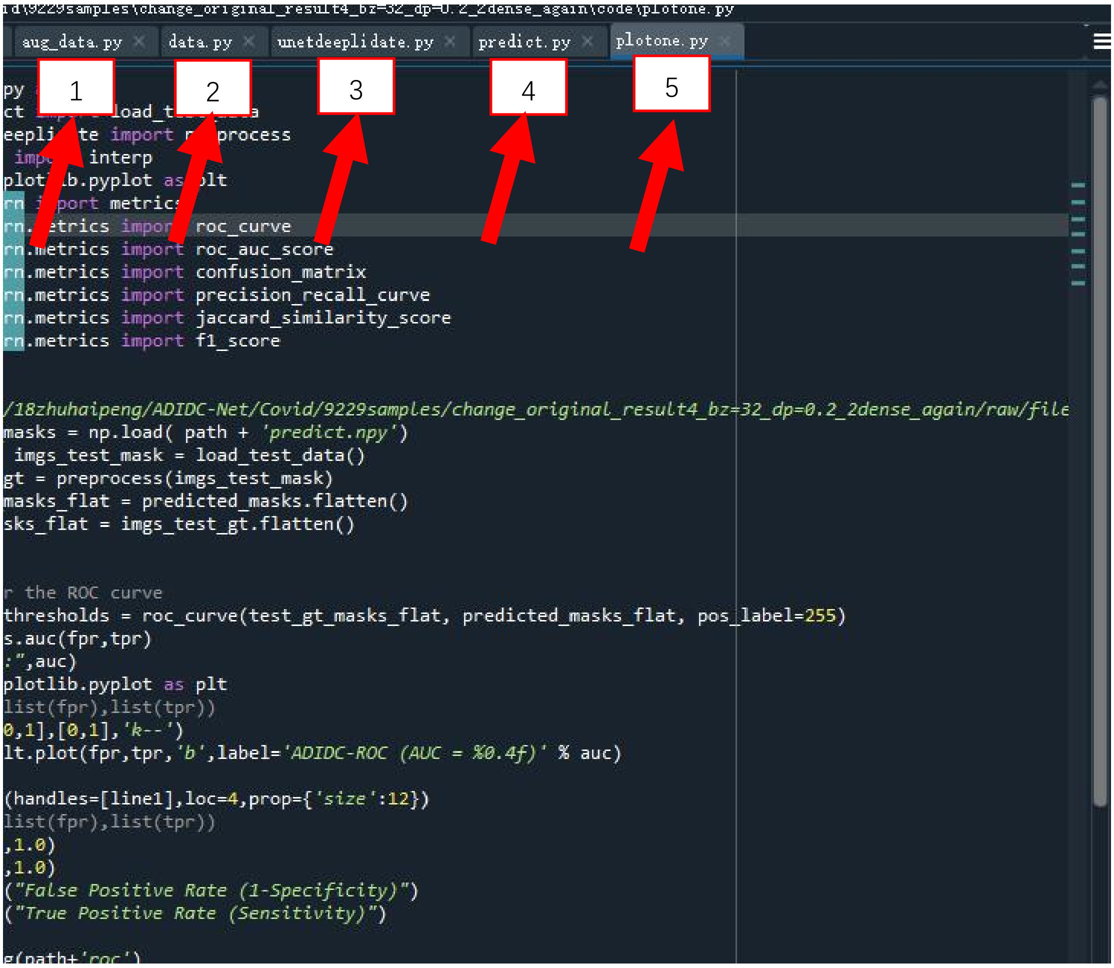

2.2 Obtaining results of ADID-NET model after executing the Python Model.

We have uploaded the test data as an attachment to PeerJ journal, and also uploaded the trained model to GitHub website https://github.com/jalexnoel/ADID-UNET.git.

(1) Download the test data and save it in the "raw" file.

(2) Then change the path of the “data.py” code and then run it, then test data will save as test.npy and test_mask.npy. Of course, you need to create a "file" folder in the "raw" folder. like this:

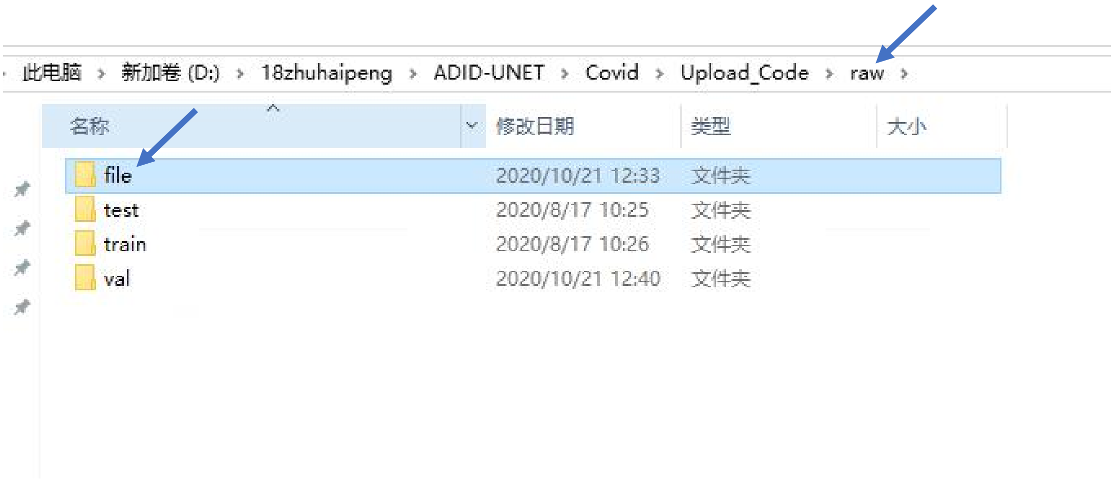

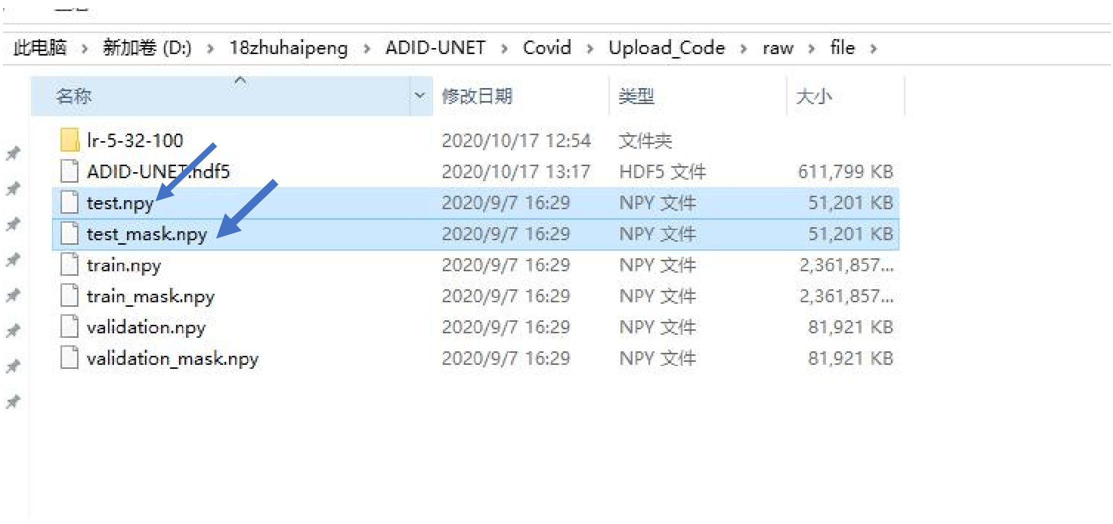

(3) We have downloaded the trained model to the "file" folder, as shown in the figure:

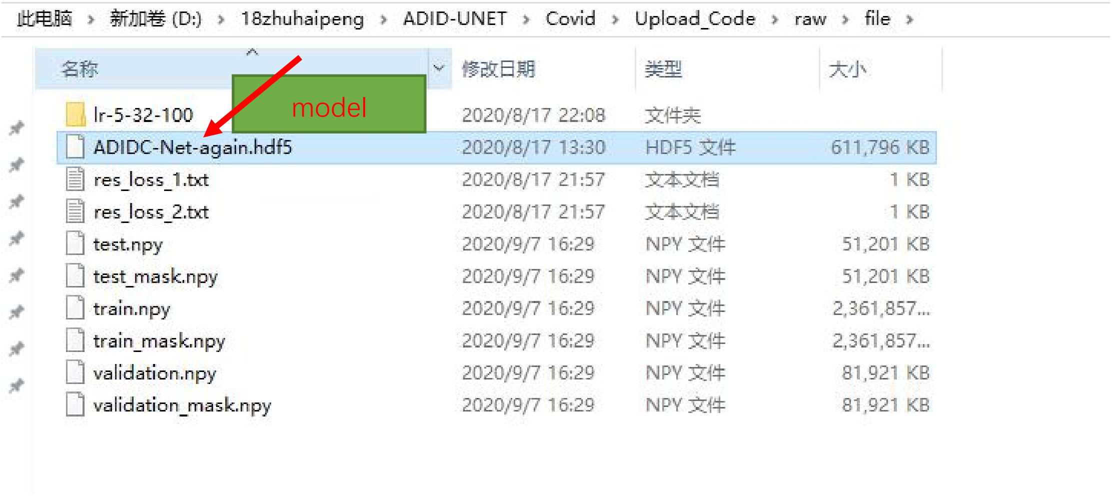

(4) Next, we just need to change the relevant path of predict.py and plotone.py. Then execute it to obtain the relevant prediction results and indicators. As shown in the figure below:

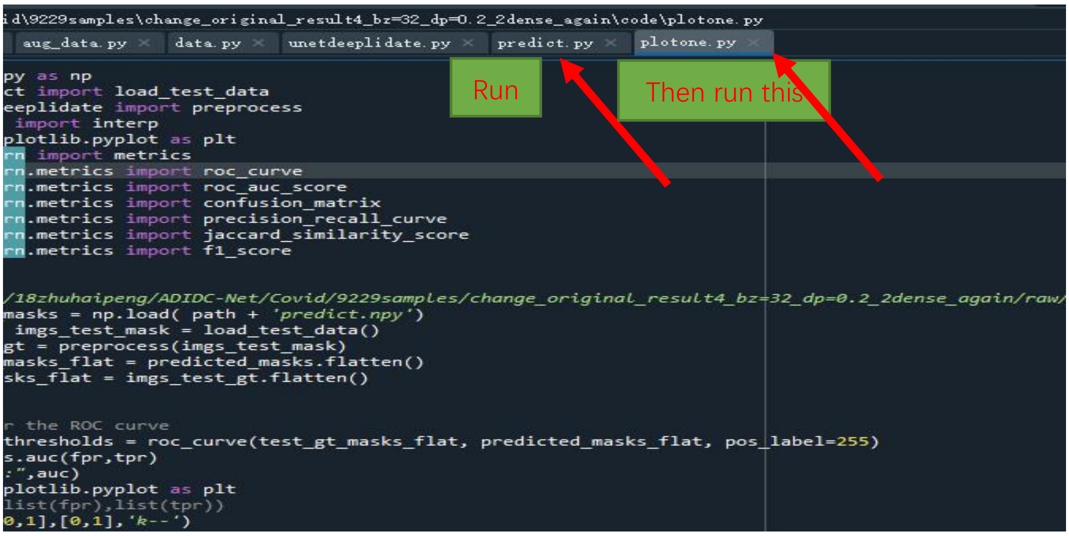


```
id\9229samples\change_original_result4_bz=32_dp=0.2_2dense_again\code\plotone.py
 aug_data.py ×   data.py ×   unetdeeplidate.py ×   predict.py ×   plotone.py ×
py as np
ct import load_test_data
eeplidate import preprocess
 import interp
plotlib.pyplot as plt
rn import metrics
rn.metrics import roc_curve
rn.metrics import roc_auc_score
rn.metrics import confusion_matrix
rn.metrics import precision_recall_curve
rn.metrics import jaccard_similarity_score
rn.metrics import f1_score

/18zhuhaipeng/ADIDC-Net/Covid/9229samples/change_original_result4_bz=32_dp=0.2_2dense_again/raw/
masks = np.load( path + 'predict.npy')
 imgs_test_mask = load_test_data()
gt = preprocess(imgs_test_mask)
masks_flat = predicted_masks.flatten()
sks_flat = imgs_test_gt.flatten()

r the ROC curve
thresholds = roc_curve(test_gt_masks_flat, predicted_masks_flat, pos_label=255)
s.auc(fpr,tpr)
:",auc)
plotlib.pyplot as plt
list(fpr),list(tpr))
0,1],[0,1],'k--')
```

Here are some predicted results and indexes:

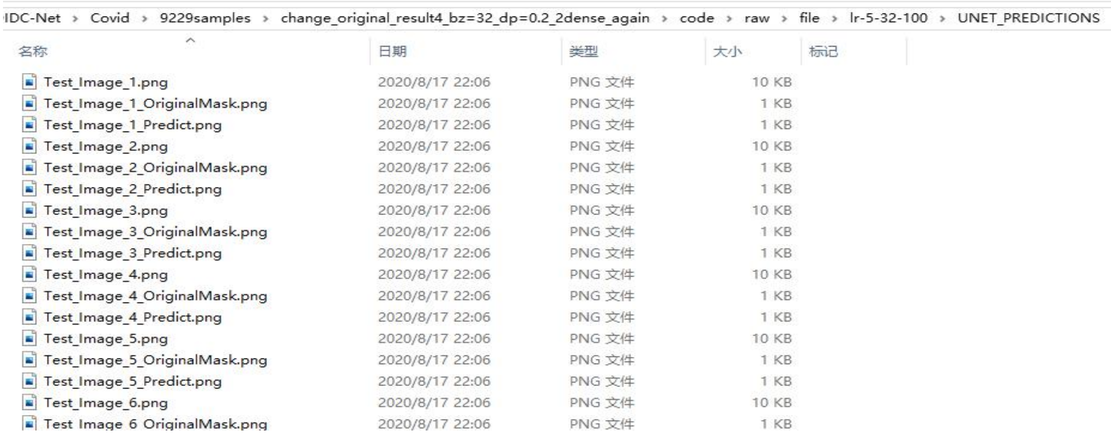

IDC-Net › Covid › 9229samples › change_original_result4_bz=32_dp=0.2_2dense_again › code › raw › file › lr-5-32-100 › UNET_PREDICTIONS

名称	日期	类型	大小	标记
Test_Image_1.png	2020/8/17 22:06	PNG 文件	10 KB	
Test_Image_1_OriginalMask.png	2020/8/17 22:06	PNG 文件	1 KB	
Test_Image_1_Predict.png	2020/8/17 22:06	PNG 文件	1 KB	
Test_Image_2.png	2020/8/17 22:06	PNG 文件	10 KB	
Test_Image_2_OriginalMask.png	2020/8/17 22:06	PNG 文件	1 KB	
Test_Image_2_Predict.png	2020/8/17 22:06	PNG 文件	1 KB	
Test_Image_3.png	2020/8/17 22:06	PNG 文件	10 KB	
Test_Image_3_OriginalMask.png	2020/8/17 22:06	PNG 文件	1 KB	
Test_Image_3_Predict.png	2020/8/17 22:06	PNG 文件	1 KB	
Test_Image_4.png	2020/8/17 22:06	PNG 文件	10 KB	
Test_Image_4_OriginalMask.png	2020/8/17 22:06	PNG 文件	1 KB	
Test_Image_4_Predict.png	2020/8/17 22:06	PNG 文件	1 KB	
Test_Image_5.png	2020/8/17 22:06	PNG 文件	10 KB	
Test_Image_5_OriginalMask.png	2020/8/17 22:06	PNG 文件	1 KB	
Test_Image_5_Predict.png	2020/8/17 22:06	PNG 文件	1 KB	
Test_Image_6.png	2020/8/17 22:06	PNG 文件	10 KB	
Test_Image_6_OriginalMask.png	2020/8/17 22:06	PNG 文件	1 KB	

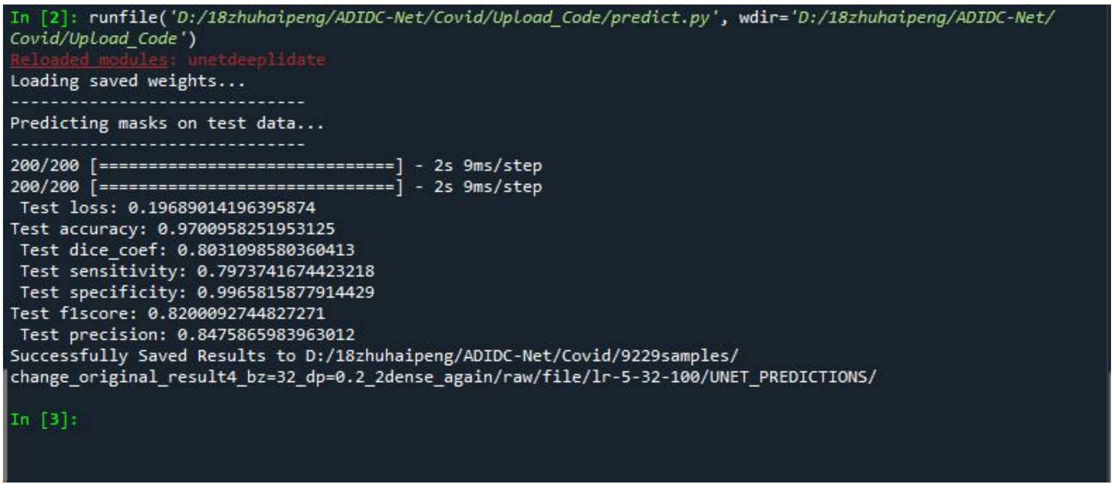

```
In [2]: runfile('D:/18zhuhaipeng/ADIDC-Net/Covid/Upload_Code/predict.py', wdir='D:/18zhuhaipeng/ADIDC-Net/
Covid/Upload_Code')
Reloaded modules: unetdeeplidate
Loading saved weights...
------------------------------
Predicting masks on test data...
------------------------------
200/200 [==============================] - 2s 9ms/step
200/200 [==============================] - 2s 9ms/step
 Test loss: 0.19689014196395874
Test accuracy: 0.9700958251953125
 Test dice_coef: 0.8031098580360413
 Test sensitivity: 0.7973741674423218
 Test specificity: 0.9965815877914429
Test f1score: 0.8200092744827271
 Test precision: 0.8475865983963012
Successfully Saved Results to D:/18zhuhaipeng/ADIDC-Net/Covid/9229samples/
change_original_result4_bz=32_dp=0.2_2dense_again/raw/file/lr-5-32-100/UNET_PREDICTIONS/

In [3]:
```

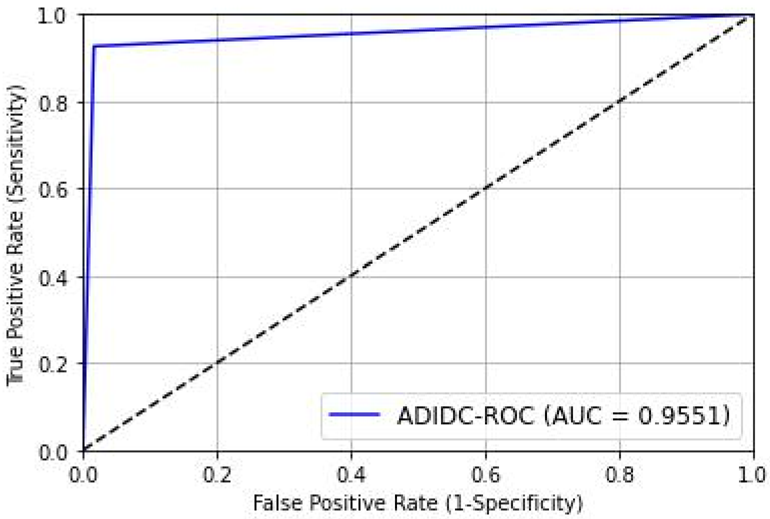

2.3 Information about the Matlab Scripts used to analyze the indicators of S_m, E_α and MAE

We can get other indicators of S_m, E_α and MAE following the "Matlab-Code" folder:

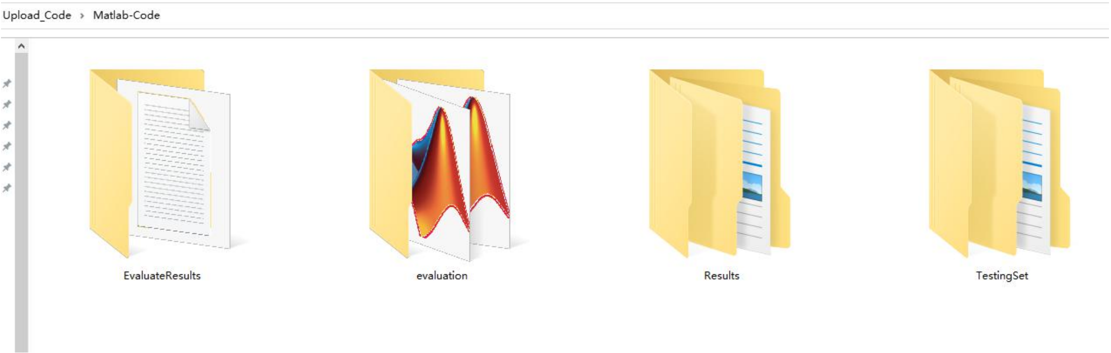

There are some Subfolders in the "EvaluateResults" folder, as shown below. The subfolders just are used to save the values after running the "main.m" code.

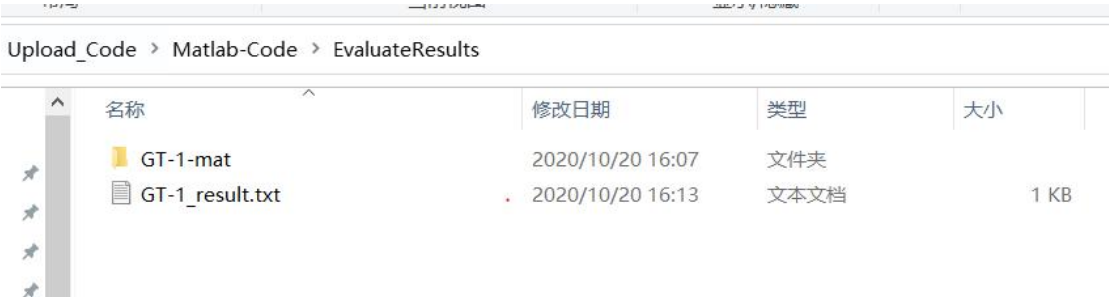

The Matlab scripts in the "evaluation" folder, as shown in the flowing figures:

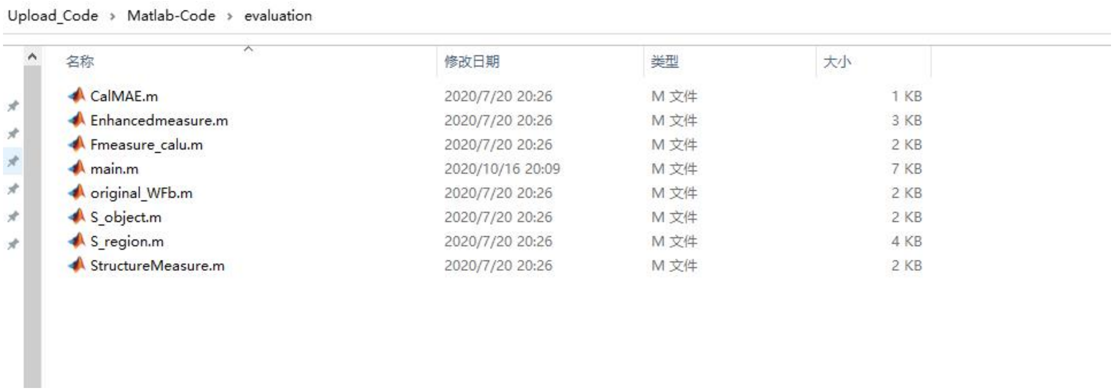

Please use "main.m" to get the evaluation results as shown below

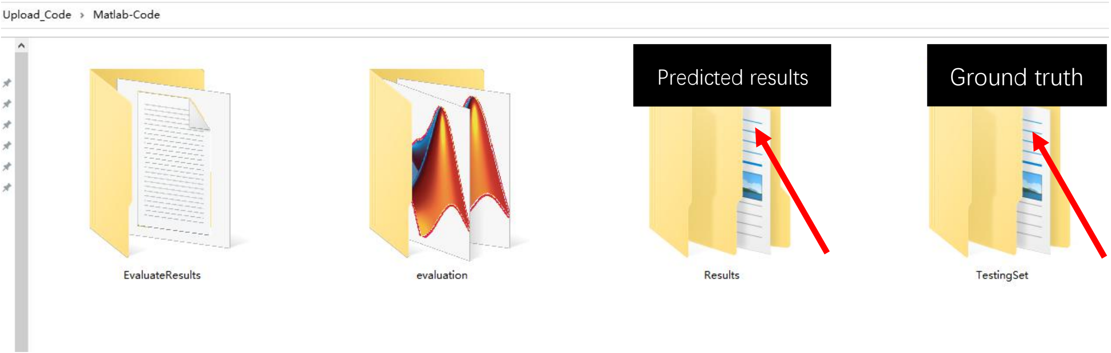

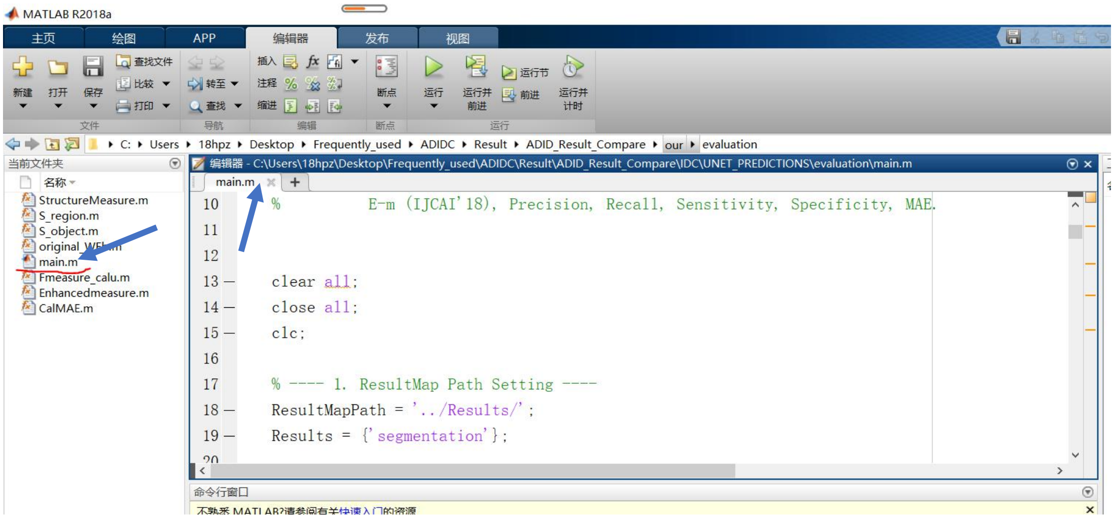

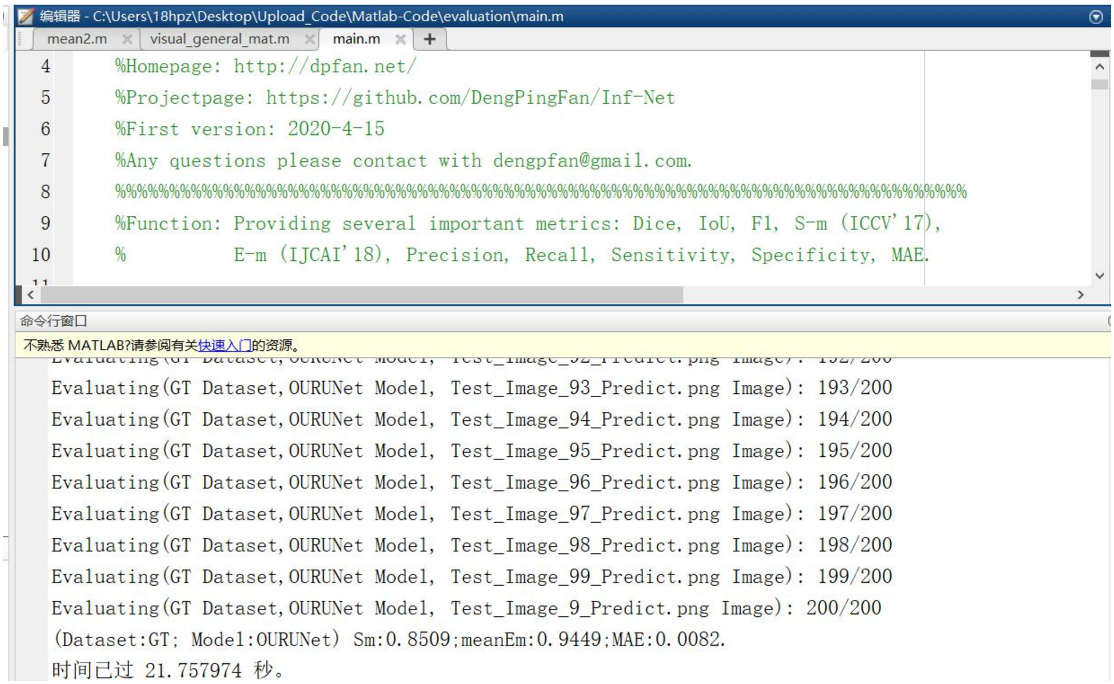
编辑器 - C:\Users\18hpz\Desktop\Upload_Code\Matlab-Code\evaluation\main.m

mean2.m | visual_general_mat.m | main.m

```
%Homepage: http://dpfan.net/
%Projectpage: https://github.com/DengPingFan/Inf-Net
%First version: 2020-4-15
%Any questions please contact with dengpfan@gmail.com.
%%%%%%%%%%%%%%%%%%%%%%%%%%%%%%%%%%%%%%%%%%%%%%%%%%%%%%%%%%%%%%%%%%%%%%%%%%%%%%%%
%Function: Providing several important metrics: Dice, IoU, F1, S-m (ICCV'17),
%          E-m (IJCAI'18), Precision, Recall, Sensitivity, Specificity, MAE.
```

命令行窗口

不熟悉 MATLAB?请参阅有关快速入门的资源。

```
Evaluating(GT Dataset,OURUNet Model, Test_Image_93_Predict.png Image): 193/200
Evaluating(GT Dataset,OURUNet Model, Test_Image_94_Predict.png Image): 194/200
Evaluating(GT Dataset,OURUNet Model, Test_Image_95_Predict.png Image): 195/200
Evaluating(GT Dataset,OURUNet Model, Test_Image_96_Predict.png Image): 196/200
Evaluating(GT Dataset,OURUNet Model, Test_Image_97_Predict.png Image): 197/200
Evaluating(GT Dataset,OURUNet Model, Test_Image_98_Predict.png Image): 198/200
Evaluating(GT Dataset,OURUNet Model, Test_Image_99_Predict.png Image): 199/200
Evaluating(GT Dataset,OURUNet Model, Test_Image_9_Predict.png Image): 200/200
(Dataset:GT; Model:OURUNet) Sm:0.8509;meanEm:0.9449;MAE:0.0082.
时间已过 21.757974 秒。
```

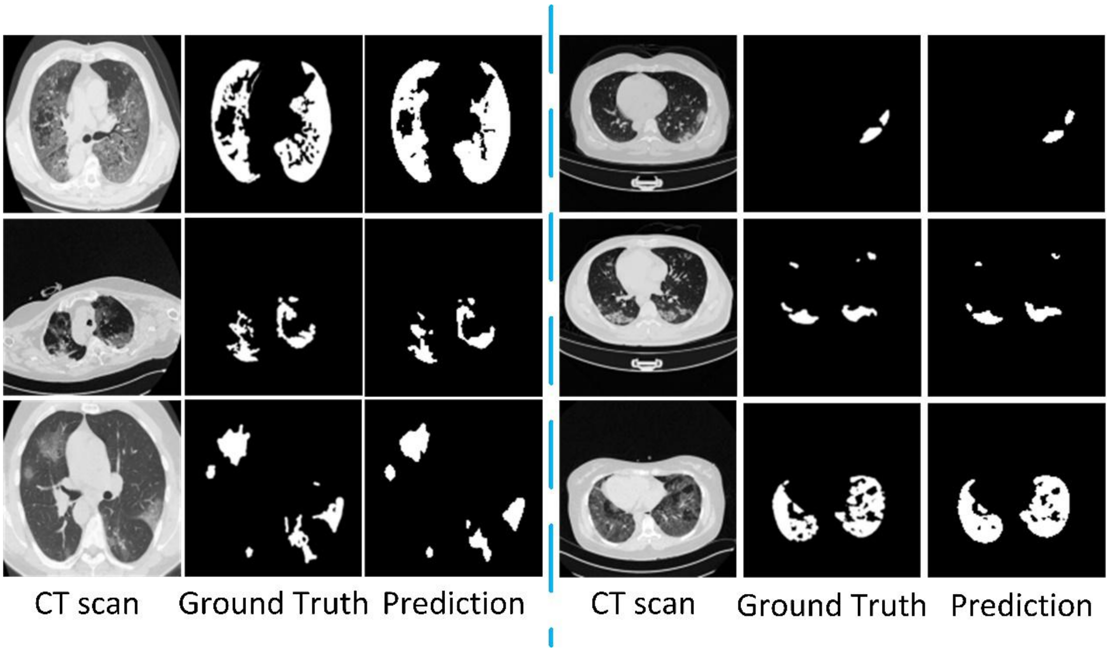

Our results are obtained through experiments and believe by submitting the codes and the usage document our works would add information to existing literature. If you have any questions, please contact us through email:

Mr. Haipenng at 18hpzhu@stu.edu.cn
Or
Dr. Alex Noel Joseph Raj jalexnoel@stu.edu.cn.
